# The older, the less potential benefit for type 2 diabetes from weight control

**DOI:** 10.1186/s12877-022-02979-8

**Published:** 2022-04-20

**Authors:** Qi Zhou, Jie Sun, Zhu Wu, Wenbin Wu, Xianbo Zhang, Qi Pan, Haimei Qi, Huiping Yuan, Hong Shi, Suyan Cao, Ze Yang, Xiaoxia Wang, Liang Sun

**Affiliations:** 1 The Key Laboratory of Geriatrics, Beijing Institute of Geriatrics, Institute of Geriatric Medicine, Chinese Academy of Medical Sciences, Beijing Hospital/National Center of Gerontology of National Health Commission, Beijing, 100730 People’s Republic of China; 2 Graduate School of Peking Union Medical College, Chinese Academy of Medical Sciences, Beijing, 100730 People’s Republic of China; 3 Geriatrics Department, Beijing Hospital, National Center of Gerontology, Institute of Geriatric Medicine, Chinese Academy of Medical Sciences, Beijing, 100730 People’s Republic of China; 4 Department of Endocrinology, Beijing Hospital, National Center of Gerontology, Institute of Geriatric Medicine, Chinese Academy of Medical Sciences, Beijing, 100730 People’s Republic of China; 5 Department of Medical and Health, Beijing Hospital, National Center of Gerontology, Institute of Geriatric Medicine, Chinese Academy of Medical Sciences, Beijing, 100730 People’s Republic of China; 6 Health Management Center, Beijing Hospital, National Center of Gerontology, Institute of Geriatric Medicine, Chinese Academy of Medical Sciences, Beijing, 100730 People’s Republic of China; 7 The NHC Key laboratory of Drug Addiction Medicine, Kunming Medical University, Kunming, 650032 China

**Keywords:** Geriatric care, The elderly, Obesity, Type 2 diabetes mellitus

## Abstract

**Background:**

Although moderate weight loss improves outcomes of type 2 diabetes mellitus (T2DM) in young and middle-aged adults, there is a lack of high-quality evidence to support the strong relationship between obesity and T2DM in older people. This study aims to investigate whether the association of obesity with T2DM changes with aging.

**Methods:**

In this cross-sectional study, we recruited 63,180 Chinses and US subjects from 3 datasets. Subjects were divided into young & middle-aged (≤59 years), young-old (60–75 years), and old-old (≥75 years). Logistic regression was used to determine the odds ratio (OR) and 95% confidence intervals (95% CI) for the association between obesity and T2DM, stratified by common confounders. A sliding-window based algorithm and restricted cubic splines were used to smoothly estimate the changes with aging.

**Results:**

The OR (95% CI) for the associations between general obesity and T2DM were decreased from the young & middle-aged group (OR, 5.91; 95% CI, 5.33–6.56) to the young-old group (OR, 3.98; 95% CI, 3.56–4.45) and then to the old-old group (OR, 3.06; 95% CI, 2.57–3.66). The trend for this reduced association with aging persisted after stratification by obesity type, region, gender, recruiting time, hypertension, and hyperlipidemia in both Chinese and Americans. We also identified a weakened gender disparity for this association between the young & middle-aged subjects (*P* for disparity < 0.001) and the old-old group (*P* for disparity = ~ 0.36).

**Conclusions:**

The obesity-T2DM association is clearly reduced with aging, which indicates that the elderly may gain fewer potential benefits in weight lose than the younger patients. Considering this attenuated association, as well as the increased incidence of geriatric syndrome in the elderly, clinicians should comprehensively balance the benefits and side effects of weight loss in geriatric T2DM interventions.

**Supplementary Information:**

The online version contains supplementary material available at 10.1186/s12877-022-02979-8.

## Background

Type 2 diabetes mellitus (T2DM) represents an important health, social and economic burden in the elderly population, that affects approximately 25 and 30% of elderly subjects in America and China, respectively [1, 2]. Obesity is a driven factor of diabetes [3, 4]; therefore, weight management has been harnessed in the prevention and treatment of T2DM. Most international guidelines recommend that patients with T2DM and overweight or obesity should achieve and maintain modest weight loss by dietary changes, physical activity, and behavioral therapy [5, 6]. The Look AHEAD trial provides evidence that weight loss of just 5–10% significantly reduced the odds of 4 years achieving T2DM and clinical related metabolic disorders, such as increased blood pressure (BP), triglycerides (TG), and decreased high-density lipoprotein cholesterol (HDL-c) [7, 8].

However, clinicians often are reluctant to recommend geriatric obesity interventions because, in the elderly, lower weight has also been associated with increased risk of certain adverse events like mortality [9]. The prevalence of sarcopenic obesity is known to be higher in the elderly [10]. Thus, intensive weight loss relying on caloric restriction could potentially worsen sarcopenia, osteoporosis, and nutrition deficits; these factors could lead to falls and fractures in elderly subjects [11, 12]. The “Obesity Paradox” is another reason for the difficulties of treatment of geriatric obesity that the appropriate elevated body mass index (BMI) between 25 ~ 30 kg/m^2^ seems to be protective for the elderly and was associated with a lower risk of mortality in older residents of nursing homes [13, 14]. The relationship between BMI and mortality in the elderly was reported to be U-shaped or reverse J-shaped, whereby the risk of mortality did not increase until extreme BMI values over 35 to 40 kg/m^2^ [13]. The effective management of older patients with diabetes requires an emphasis on safety, therefore, clinicians should fully consider the controversial issues surrounding the benefits and harm of T2DM and obesity management, from lifestyle to medications [15, 16].

There is a lack of high-quality evidence to support the efficacy of weight loss on T2DM in older people [15]. Sex hormones, body composition, as well as lifestyle, all change significantly with aging. These changes increase the heterology of health status and caused geriatric syndromes, thus complicates the management of T2DM in older adults [17]. A recent systematic report claimed that the current evidence supporting the use of obesity interventions in the geriatric population to improve physical function was of low to moderate quality [18]. There is also confusion as to whether the regional and sexual disparities underlying the relationship between obesity and T2DM change with aging because the distribution of fat and muscle showed variation across different genders, regions, and ages [19].

To address these gaps in knowledge, we investigated the association between geriatric obesity and T2DM by enrolling 63,180 subjects from one American and two Chinese datasets. We also investigated sexual and racial disparities for this association, and how disparities changed when comparing between younger and older subjects.

## Material and methods

### Data sources

This was a cross-sectional study based on an American dataset (from a national survey), a northern Chinese dataset (in Beijing city), and a southern Chinese dataset (in Guangdong Province): 1) the American data was collected from the National Health and Nutrition Examination Survey (NHANES, 1999–2018, including 10 circles), which finally included 48,072 subjects (men, 48.4%) aged 18 to 85 years. 2) The Guangdong Chinese dataset was obtained from the Guangdong Gut Microbiome Project (GGMP) [20], which included 6914 subjects (men,41.1%) aged 18 to 97 years and enrolled in 2018. 3) the Beijing Chinese dataset was obtained by annual physical examinations of the community population in Beijing Hospital in 2008, which included 8149 subjects (men, 44.9%) aged 18 to 83 years. The three studies were approved by the Institutional Review Board of the Centres for Disease Control and Prevention of America, the Ethical Review Committee of the Chinese Centre for Disease Control and Prevention, and the Institutional Ethics Committee at Beijing Hospital, respectively.

All the enrolled subjects were required to have information including age, sex, fast plasma glucose (FPG), BMI and/or waist circumference, and those who were reported to have cancer or malignancy tumor were excluded.

Subjects were divided into young & middle-aged adults (≤59 years) and the elderly (≥60 years), who were sub-grouped into young-old (60 ~ 74 years), and the old-old (≥75 years).

### Definitions of obesity and T2DM

BMI and waist circumference were used to define general obesity and abdominal obesity, respectively. For American subjects, general obesity was assessed by BMI ≥ 30 kg/m^2^ and overweight was assessed by BMI ≥ 25 kg/m^2^ but < 30 kg/m^2^; abdominal obesity was defined with a waist circumference ≥ 102 cm in men and ≥ 88 cm in women [21]. For Chinese subjects, general obesity was assessed by BMI ≥ 28 kg/m^2^ and overweight was assessed by BMI ≥ 24 kg/m^2^ but < 28 kg/m^2^; abdominal obesity was defined with a waist circumference ≥ 90 cm in men and ≥ 85 cm in women [22].

Subjects who have one of the following criteria were defined as T2DM according to the American Diabetes Association [23]: self-reported doctor-diagnosed diabetes, fasting plasma glucose ≥7.0 mmol/L, 2-h plasma glucose ≥11.1 mmol/L, or HbA1c ≥6.5%.

### Statistical analyses

We reported characteristics of the subjects over age categories and stratified by sex and region. Descriptive statistics were summarized as mean ± standard deviation (SD) for continuous variables, or an absolute number with percentages for categorical variables.

A sliding-window-based algorithm (SWAN) was developed to sample and analyse trajectories or trends of factors across aging for cross-sectional studies [24]. SWAN collected a batch of data in an age window of 5 to 10 years. The window slid from 20 to 80 years with an increment of 1 year. This algorithm was used for data sampling in the following progress: 1) calculating the mean value of BMI, waist circumference, and FPG in the aging; 2) estimating how risks of T2DM for obese subjects changes from 20 to 80 years old; 3) comparing differences of odds ratio (OR) between men and women in young & middle-aged adults, the young-old elderly, and old-old elderly, respectively.

Correlations between BMI, waist circumference, and age were investigated by the Pearson correlation test. Wilcoxon test was used to test the significance of Pearson coefficients in subjects before and after 60 years old.

Logistic regression stratified by sex, region, and age was used to estimate the OR and 95% confidence intervals (95%CI) for the associations between T2DM and overweight, general obesity, and abdominal obesity, in which subjects of normal weight were compared as a referent.

Restricted cubic splines were fitted in the logistic regression to flexibly study the association between BMI and T2DM occurrence, in which BMI of 30 kg/m^2^ was set as a referent. ΔBMI was calculated when ORs decreased from 1.0 to 0.5 (ΔBMI = BMI_OR1.0_ – BMI_OR0.5_) which represented the risk of T2DM was reduced by half.

We stratified sex in the logistic regression with SWAN to investigate the gender disparity in three age groups. Firstly, ORs for women and men were respectively estimated in each age window based on SWAN. Secondly, a pair of ORs for men and women in each window was defined as a “paired comparison”. Finally, all the comparisons were collected, and the difference of ORs between men and women was tested by the Wilcoxon test, respectively in the young & middle-aged group, the young-old, and the old-old group.

All data analyses and plots were performed using R × 64 4.0.0 for windows. Statistical significance was determined by a two-tailed test with a cut-off value of *P* ≤ 0.05.

### Sensitivity analyses

Blood pressure (BP), including systolic blood pressure (SBP) and diastolic blood pressure (DBP), total cholesterol (TC); TG; HDL-c, and low-density lipoprotein cholesterol (LDL-c) were stratified in logistic models. Abnormalities in these factors were defined by the following criteria: increased BP, SBP/DBP ≥ 130/85 mmHg; increased TC, ≥5.17 mmol/L; increased TG, ≥ 1.70 mmol/L; reduced HDL-c, < 1.03 mmol/L for men and < 1.30 mmol/L for women; increased LDL-c, > 3.3 mmol/L. Recruiting time (circles) for NHANES were also stratified in logistic models; one circle lasted for 2 years in the NHANES project.

## Results

### Descriptive characteristics

The characteristics of the three datasets are presented in Table 1. In total, 63,180 adult individuals were surveyed. Chinese subjects had a lower prevalence of obesity and T2DM than American subjects. Approximately 10.2–14.5% of the Chinese subjects had general obesity, 23.8–30.0% were abdominal obesity, and 5.5–8.0% had T2DM; In the American subjects, 35.4% had general obesity, 53.2% had abdominal obesity, and 10.8% had T2DM.

**Table 1:** Characteristics of participants, stratified by region, age, and sex (*N* = 63,135)

	Overall			Young & middle-aged (< 60 years)			The elderly (≥ 60 years)		
	Total	Women	Men	Total	Women	Men	Overall	Women	Men
Beijing Chinese									
n	8149	3372	4777	6120	2667	3453	2029	705	1324
Age (year)	48.2 ± 15.4	41.1 ± 10.1	41.5 ± 9.8	41.1 ± 10.1	40.7 ± 10.6	41.5 ± 9.8	69.5 ± 5.8	69.7 ± 6.1	69.4 ± 5.7
BMI (kg/m^2^)	24.4 ± 3.6	22.9 ± 3.4	25.4 ± 3.2	24.3 ± 3.6	22.6 ± 3.3	25.6 ± 3.3	24.6 ± 3.3	24.1 ± 3.6	24.8 ± 3.1
Waist circumference (cm)	83.1 ± 10.5	76.7 ± 9.5	87.7 ± 8.6	82.2 ± 10.7	75.1 ± 8.6	87.7 ± 8.6	86 ± 9.5	82.8 ± 10.1	87.8 ± 8.7
FPG (mmol/L)	5.4 ± 1.2	5.2 ± 1.0	5.5 ± 1.3	5.3 ± 1.1	5.1 ± 0.8	5.4 ± 1.2	5.8 ± 1.4	5.7 ± 1.4	5.9 ± 1.4
Underweight, n, (%)	282 (3.5)	218 (6.5)	64 (1.3)	227 (3.7)	186 (7)	41 (1.2)	55 (2.7)	32 (4.5)	23 (1.7)
Overweight, n, (%)	3224 (40.8)	906 (26.9)	2318 (48.5)	2359 (38.5)	655 (24.6)	1704 (49.3)	865 (42.6)	251 (35.6)	614 (46.4)
General obesity, n, (%)	1181 (14.5)	269 (8.0)	912 (19.1)	886 (14.4)	168 (6.3)	718 (20.8)	295 (14.5)	101 (14.3)	194 (14.7)
Abdominal obesity, n, (%)	2504 (30.8)	648 (19.2)	1956 (40.9)	1779 (29.1)	372 (13.9)	1407 (40.7)	825 (40.7)	276 (39.1)	549 (41.5)
T2DM, n, (%)	451 (5.5)	98 (2.9)	353 (7.4)	234 (3.8)	45 (1.7)	189 (5.5)	217 (10.7)	53 (7.5)	164 (12.4)
Guangdong Chinese									
n	6914	3812	3102	4508	2595	1913	2406	1217	1189
Age (year)	52.7 ± 14.7	51.9 ± 14.5	53.7 ± 14.9	44.5 ± 10.5	44.4 ± 10.3	44.5 ± 10.7	68.3 ± 6.7	68.1 ± 6.8	68.5 ± 6.5
BMI (kg/m^2^)	23.4 ± 3.5	23.4 ± 3.6	23.3 ± 3.4	23.5 ± 3.5	23.4 ± 3.6	23.6 ± 3.5	23.1 ± 3.5	23.4 ± 3.6	22.8 ± 3.4
Waist circumference (cm)	80.3 ± 9.9	78.8 ± 9.7	82.2 ± 9.9	79.6 ± 9.9	77.7 ± 9.5	82.2 ± 9.8	81.6 ± 10	81.2 ± 9.9	82.0 ± 10.8
FPG (mmol/L)	5.6 ± 1.7	5.6 ± 1.7	5.7 ± 1.6	5.5 ± 1.6	5.5 ± 1.6	5.6 ± 1.5	5.9 ± 1.8	5.9 ± 2	5.8 ± 1.7
Underweight, n, (%)	468 (6.7)	260 (6.8)	208 (6.7)	272 (6.0)	172 (6.6)	100 (5.2)	196 (8.1)	88 (7.2)	108 (9.1)
Overweight, n, (%)	2060 (29.8)	1110 (29.1)	950 (30.6)	1367 (30.3)	749 (28.9)	618 (32.3)	693 (28.8)	361 (29.7)	332 (27.9)
General obesity, n, (%)	707 (10.2)	417 (10.9)	290 (9.3)	489 (10.8)	284 (10.9)	205 (10.7)	218 (9.1)	133 (10.9)	85 (7.1)
Abdominal obesity, n, (%)	1647 (23.8)	965 (25.3)	682 (22.0)	970 (21.5)	555 (21.4)	415 (21.7)	677 (28.1)	410 (33.7)	267 (22.5)
T2DM, n, (%)	553 (8.0)	278 (7.3)	255 (8.2)	277 (6.1)	144 (5.6)	133 (7)	256 (10.7)	134 (11.0)	122 (10.3)
American									
n	48,072	23,251	24,821	34,803	17,985	16,818	13,269	6836	6433
Age (year)	45.7 ± 19.2	45.7 ± 18.7	45.8 ± 18.5	36.5 ± 12.3	36.3 ± 12.2	36.7 ± 12.5	69.9 ± 7.3	70.2 ± 7.4	69.7 ± 7.1
BMI (kg/m^2^)	28.7 ± 6.8	29.2 ± 7.5	28.3 ± 6.1	28.7 ± 7.1	29.1 ± 7.8	28.2 ± 6.3	29.0 ± 6.1	29.5 ± 6.7	28.5 ± 5.4
Waist circumference (cm)	97.6 ± 16.3	96.0 ± 16.6	99.3 ± 16.0	96.1 ± 16.8	94.9 ± 17.2	97.5 ± 16.2	101.5 ± 14.414.4	99.0 ± 14.3	104.2 ± 13.9
FPG (mmol/L)	5.9 ± 2.0	5.8 ± 1.9	6.1 ± 2.1	5.7 ± 1.8	5.5 ± 1.7	5.9 ± 1.9	6.5 ± 2.3	6.4 ± 2.3	6.7 ± 2.3
Underweight, n, (%)	896 (1.9)	550 (2.2)	346 (1.5)	744 (2.1)	468 (2.6)	276 (1.6)	152 (1.1)	82 (1.2)	70 (1.1)
Overweight, n, (%)	15,833 (32.9)	7085 (28.5)	8748 (37.6)	10,905 (31.3)	4837 (26.9)	6068 (36.1)	4928 (37.1)	2248 (32.9)	2680 (41.7)
General obesity, n, (%)	17,015 (35.4)	9655 (38.9)	7360 (31.7)	12,125 (34.8)	6861 (38.1)	5264 (31.3)	4890 (36.9)	2794 (40.9)	2096 (32.6)
Abdominal obesity, n, (%)	24,796 (53.2)	15,711 (65.6)	9805 (40.1)	16,477 (48.5)	10,704 (61.2)	5773 (35.0)	8319 (66.0)	5007 (77.7)	3312 (53.7)
T2DM, n, (%)	6818 (14.2)	3284 (13.2)	3534 (15.2)	2337 (6.7)	1085 (6.0)	1252 (7.4)	3080 (23.2)	1446 (21.2)	1634 (25.4)

*BMI* body mass index, *FPG* fast plasma glucose, *T2DM* type 2 diabetes mellitus

The prevalence of T2DM, general obesity, and abdominal obesity were generally higher in the elderly compared with the young & middle-aged subjects; this was the case across all regions. However, the opposite trend was observed in Chinese men; older subjects had a lower prevalence of general obesity than young & middle-aged adults (20.8% vs 14.7% in the Beijing Chinese dataset; 10.7% vs 7.1% in the Guangdong Chinese dataset).

Gender differences in the prevalence of T2DM, general obesity, and abdominal obesity, were also observed. T2DM individuals were more likely to be males in all regions. The prevalence of general obesity and abdominal obesity was more likely to increase in females in both Guangdong Chinese and American datasets.

### Correlations between BMI, waist circumference, FPG, and age

**Fig. 1:**
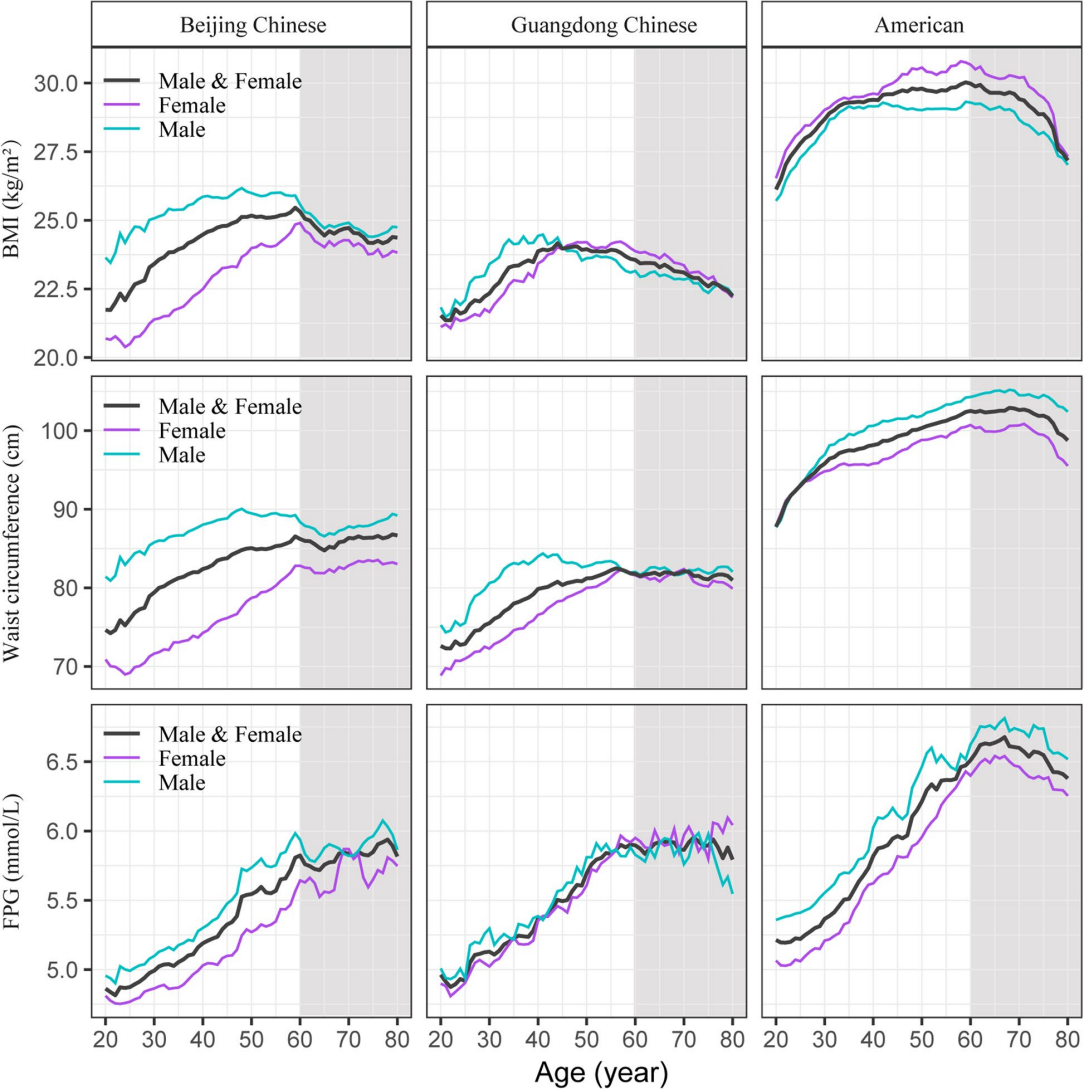
The trajectory of BMI, waist circumference and fasting plasma glucose with increasing age. The mean values of BMI, waist circumference, and fasting plasma glucose were estimated by the SWAN algorithm with stratification for sex and region

Figure 1 shows the changes in BMI, waist circumference, and FPG, as age increases. BMI, waist circumference, and FPG changed in a “U/J” shape with increased age. The shapes were similar between males and females but showed clear disparities between regions. Of note, for Chinese subjects, BMI or waist circumference decreased from middle-aged adults to the elderly (≥ 60 years), whereas FPG kept at a stable concentration. This indicated that the association between FPG and BMI or waist circumference may change with aging.

Therefore, we estimated how the correlation of FPG with BMI and waist circumference changed between young & middle-aged adults and the elderly by applying the Pearson’s correlation test (Supplementary Table 1). A significant correlation was found in all the subjects between BMI and FPG (coefficients: 0.13–0.27, P for correlation < 0.01), however, the coefficients in the elderly were significantly lower than those in young & middle-aged adults (*P* for age differences < 0.01); this trend remained unchanged when we stratified the data by region, sex, and type of obesity.

### The association between obesity and T2DM decreased with aging

**Fig. 2:**
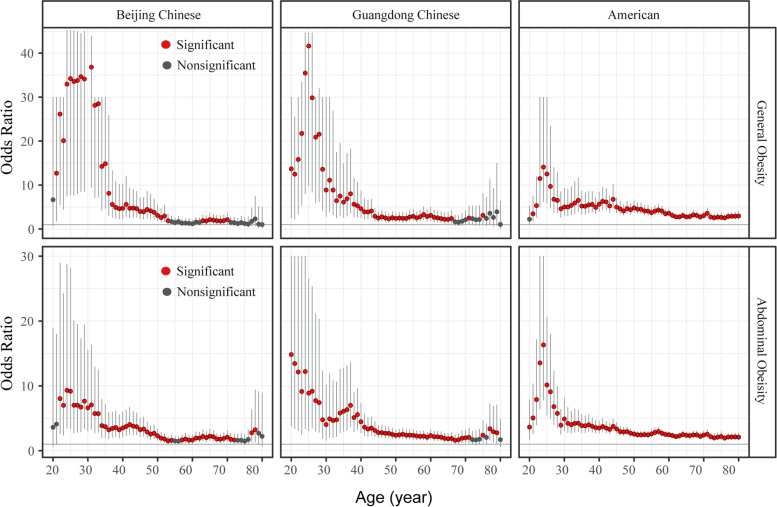
Changes of the association between obesity and T2DM with increasing age. Logistic regression analyses with the SWAN algorithm were performed with general or abdominal obesity as predictors and stratified by region. Odds ratios with 95% confidence intervals (dots with error bars) were estimated for each age window. Non-significant odds ratios are shown as black dots

To further verify the effects of aging on the associations between obesity and T2DM, we estimated how the ORs changed with increased age, as determined by the SWAN algorithm (Fig. 2). The ORs for associations between obesity and T2DM decreased from ~ 30 to 80 years in subjects with general and abdominal obesity; the association even became non-significant when age increased to 70–75 years old in Chinese subjects.

Next, we compared the ORs between young & middle-aged adults, the young-old, and the old-old (Supplementary Table 2). Consistent with the results derived by the SWAN algorithm, we found that the ORs for the association between general obesity and T2DM reduced consistently from the young & middle-aged adults (OR, 5.91; 95%CI, 5.33–6.56) to the young-old group (OR, 3.98; 95%CI, 3.56–4.45) and the old-old group (OR, 3.06; 95%CI, 2.57–3.66). This trend persisted when we stratified data by region, sex, and type of obesity. Of note, this trend was more pronounced in Chinese subjects in that the ORs were non-significant in the old-old group: the ORs (95%CI) were 1.28 (0.56–2.73) and 2.57 (0.89–6.47) in the Beijing and Guangdong Chinese old-old group, respectively. In addition, gender disparity was observed in the associations between obesity and T2DM; these disparities changed with aging (Supplementary Table 3). Considering the higher prevalence of other metabolic diseases in the elderly and the different performing time in NHANES dataset, we further stratified the data by hypertension, hyperlipidemia (Supplementary Fig. 1), and recruiting time (Supplementary Fig. 2), among which we observed the same tendencies.

**Fig. 3:**
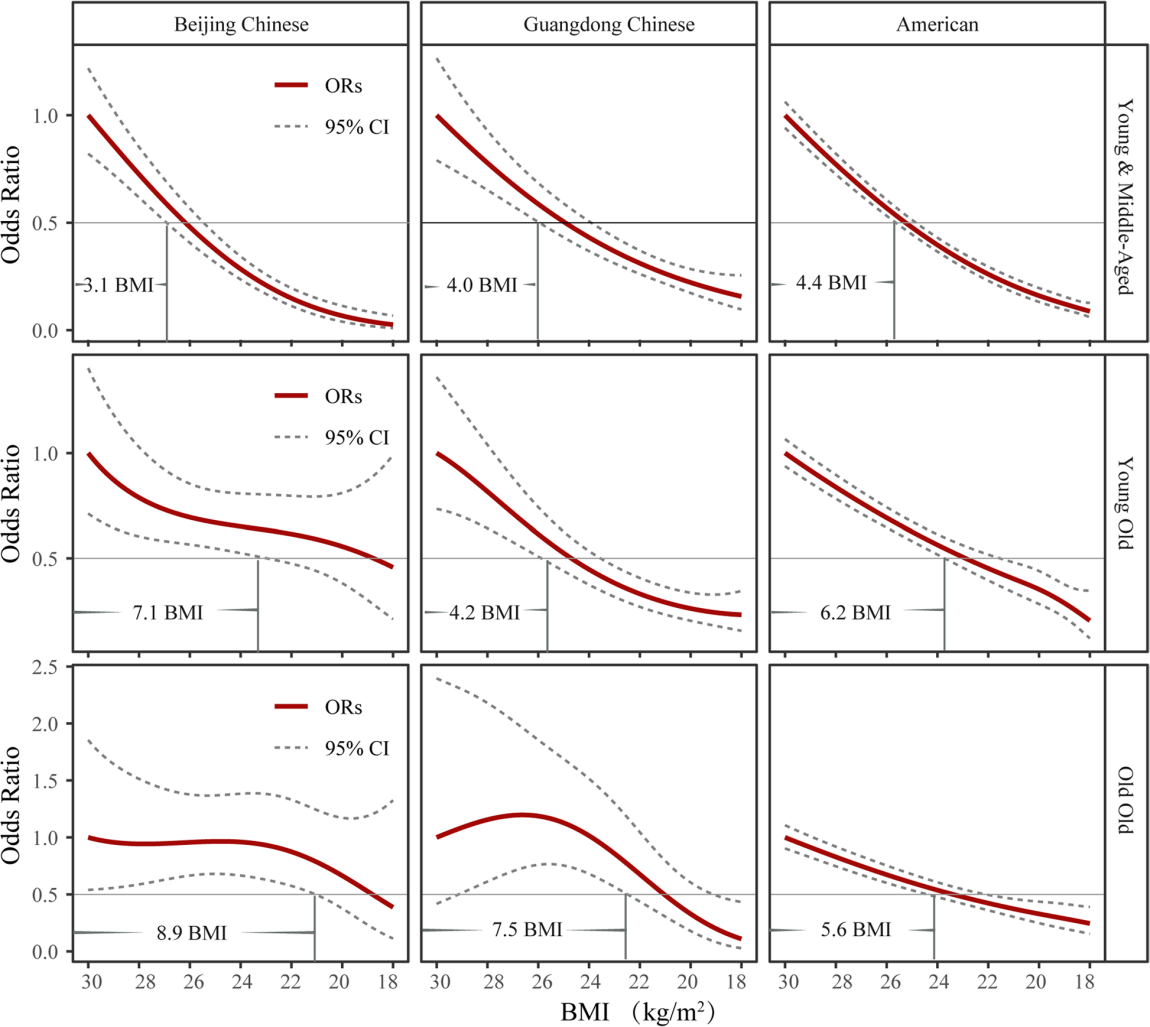
The association between BMI and T2DM. Logistic regression analyses, with restricted cubic splines, were performed with stratification for region and age. A BMI of 30 kg/m^2^ was chosen as a reference. A 50% reduction in the risk of T2DM was fitted by ΔBMI; estimates showed that the OR decreased from 1.0 to 0.5 (ΔBMI = BMI_OR1.0_ – BMI_OR0.5_). The red lines and the dotted lines represent the odds ratios and the 95% confidence intervals, respectively

To confirm the robustness of these findings, we ran an algorithm involving restricted cubic splines in logistic models to fit how the risk of T2DM decreased with reduced BMI in all age groups. A BMI of 30 kg/m^2^ was set as a reference for these models and ΔBMI represented the degree of weight loss accompanied by a reduction in the risks of T2DM (Fig. 3). A reduction of 3.1 ΔBMI was observed when the risk of T2DM was reduced by half in young & middle-aged subjects from Beijing China, while a reduction of 7.1 and 8.9 ΔBMI was observed in young-old and old-old subjects, respectively. A similar trend was observed in Beijing Chinese and American subjects, thus revealing a reduced association between obesity and T2DM in the elderly, and that the elderly may need to lose more weight than younger adults to reduce the risk of T2DM by the same extent.

### Gender disparities in associations between obesity and T2DM

**Fig. 4:**
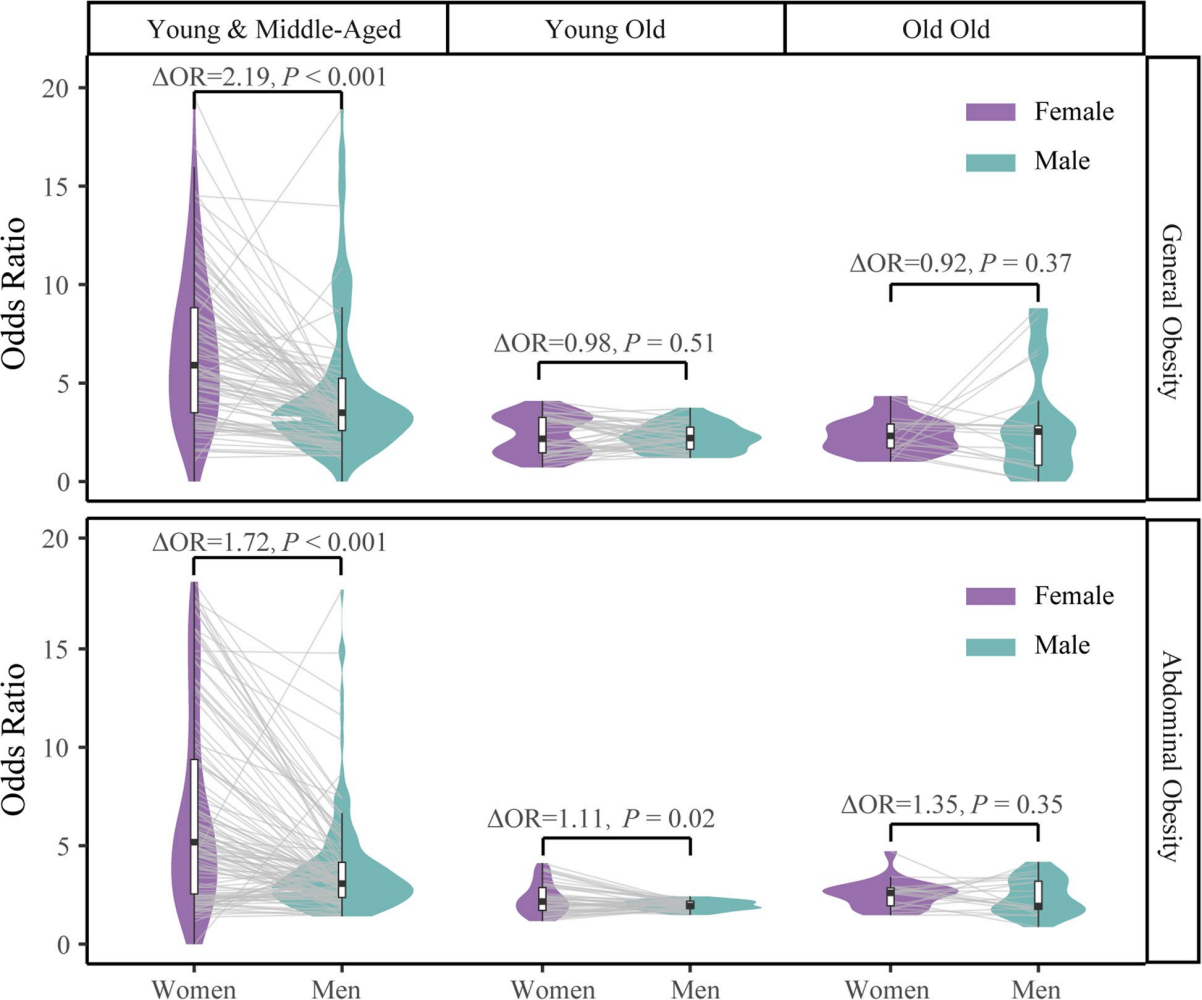
Gender disparity in the odds ratio for the association between obesity and T2DM among three age groups. The odds ratio for men and women was estimated by logistic regression and the SWAN algorithm, as stratified by age. A comparison of the odds ratios between men and women was performed for each age window and connected by grey lines. The difference of odds ratio in each age group was tested by the Wilcoxon test. ΔOR = OR _in women_/OR _in men_

To investigate how the gender differences changed with aging, we performed 189 comparisons of ORs between males and females with the SWAN (Fig. 4). In most comparisons, the ORs in women were higher than men, however, the gender difference was reduced in the elderly. With regards to the association between T2DM and general obesity, the OR in women was 2.19-fold higher than those in men in the young & middle-aged group (P for disparity < 0.001), while the ΔOR (OR in women/OR in men) reduced to 0.92–0.98 and became non-significant in the young-old group (*P* = 0.51) and old-old group (*P* = 0.37). These reductions in gender gaps were more pronounced among Chinese subjects; the ORs in men increased and even exceeded that in women (Supplementary Table 3). Similar trends were evident for abdominal obesity. Collectively, our data indicate that the association between obesity and T2DM was stronger in women than men; however, the differences between genders decreased with aging.

## Discussion

In this cross-sectional study, we found that obesity was significantly associated with an elevated prevalence of T2DM; however, this association was attenuated with aging in both Chinese and Americans. We also found the gender disparity in the association was reduced from young adults to the elderly. This is the first systematic study to investigate the interactions of aging in the associations between obesity and T2DM in both Chinese and Americans.

The association between obesity and T2DM, as well as the correlation between FPG and BMI/Waist circumference, decreased with aging and even became non-significant in the old-old group. The reasons for this reduced association are not clear, but might be explained by several mechanisms. Firstly, by selection effect; in other words, subjects with severe dysmetabolic obesity died at a younger age. Thus, people with a fat mass that is less metabolically active are left to survive to an older age. In such cases, the presence of an energy reserve that could be used in the case of acute or chronic disease could be beneficial during the geriatric period of life [25, 26]. Secondly, the power of BMI/waist circumference as the indicator of fat mass or visceral obesity may be reduced in the elderly and then affect the associations between BMI/waist circumference and T2DM. The classification of obesity by BMI /waist circumference could not reflect the variations in lean body mass, fat mass, or fluid retention [25, 27]. Fat is redistributed from the central region to the lower limbs with aging, but the absolute total body fat mass does not accumulate and even declines after 75 years old [28]. It should not be ignored that subjects over 75 years were excluded in most clinical trials since their elevated health risks in attending clinical research and relative complex health status, such as the high prevalence of geriatric syndrome [29]. Accordingly, although BMI/waist circumference is convenient items for body measurement, their cut-off value, and applicability for geriatric T2DM management, especially for individuals over 75 years, should be further investigated.

The association between obesity and T2DM was stronger in women than men; the gender disparity was also attenuated with aging. The general higher ORs in young women indicated that with the same degree of weight gain, women may have a higher risk for the development of T2DM, although men have a higher prevalence of metabolic disease at the same BMI [30, 31]. The mechanisms underlying the gender disparities of obesity that are induced by metabolic complications have yet to be elucidated; however, significant metabolic and phenotypic differences exist between genders in obesogenic environments and these are likely to play critical roles. The higher muscle mass of men, compared with women, may contribute to the lower effect of obesity on T2DM, because skeletal muscle, responsible for the majority of basal and insulin-stimulated glucose uptake, plays a critical role in regulating glucose homeostasis [32, 33]. With aging, a larger magnitude of muscle reduction occurs in men compared to women [35]; this can partially explain the reduced sexual dimorphism of the associations between obesity and T2DM in the present study.

Regional factors might potentially influence the effect of weight control on the risk of diabetes. Asians have distinct genetic factors, dietary patterns, lifestyle, life expectancy, and economic development levels compared with Americans [35, 36]. In our study, the involved Chinese subjects showed a lower prevalence of T2DM and obesity and a weaker correlation between them compared with Americans, which need further validation in high-quality datasets based on the comparable sampling methods. This requires a very careful interpretation since of different sampling methods and potential influence of life expectancies among regions. Generally, the overall life expectancies among the three groups are very close in both genders (Supplementary Table 4). It is noteworthy that obesity was no longer a risk factor for T2DM in Chinese old-old subjects (*P* for ORs > 0.05). As most recommendations of weight loss for T2DM intervention were from American and European participants [15, 16], studies in the elderly population of Asians are needed in the future, and the influence of age and regions should also be further explored.

This study has several limitations that need to be considered. First, the evidence from this cross-sectional study was not as strong as that derived from well-conducted multicentred trials. Although we performed sensitivity analyses by stratifying hypertension, hypertriglyceridemia, increased total cholesterol, reduced HDL-c, increased LDL-c, and recruiting time, it is possible that many potential confounders (including diet and physical activity) could have been lost from our analysis, thus causing bias. Adequately powered studies such as well-conducted randomized-controlled trials, cohort studies, and case-control studies should be further performed. Second, the sample size for the involved elderly Chinese subjects was smaller (*n* = 4435) compared with individuals in NHANES (*n* = 13,269), which may reduce the statistical power in the analysis of the association between obesity and T2DM, especially in the subgroups with advanced ages. Third, a selection bias existed for Chinese datasets. Only two areas were included and cannot represent the Chinese population; high-quality nation-representative surveys like NHANES should be conducted in the future.

## Conclusions

Overall, our findings suggest that the association between T2DM and obesity decreased with aging in both men and women in Chinese and American subjects. Considering this reduced association, as well as the increased risk of geriatric syndrome with weight loss, clinicians should comprehensively balance the benefits and side effects of weight loss for T2DM interventions in the elderly.

## Supplementary Information

**Additional file 1.**

## Data Availability

The data of NHANES were from https://www.cdc.gov/nchs/nhanes/index.html; the data of GGMP was obtained from the supplementary data of the published article [20].

## Abbreviations

T2DM: Type 2 diabetes mellitus
BP: Blood pressure
HDL-c: High-density lipoprotein cholesterol
TG: Triglycerides
NHANES: National Health and Nutrition Examination Survey
GGMP: Guangdong Gut Microbiome Project
FPG: Fast plasma glucose
BMI: Body mass index
SD: Standard deviation
SWAN: Sliding-window-based algorithm
OR: Odds ratio
CI: Confidence interval
SBP: Systolic blood pressure
DBP: Diastolic blood pressure
TC: Total cholesterol
LDL-c: Low-density lipoprotein cholesterol
