# Supplementary material for: The older, the less potential benefit for type 2 diabetes from weight control
Source: BMC Geriatr. 2022 Apr 20;22:346. doi: 10.1186/s12877-022-02979-8 (PMC9022304; doi:10.1186/s12877-022-02979-8)
Supplement: Supplementary file 1 — Additional file 1. [file 12877_2022_2979_MOESM1_ESM.docx]

**Supplementary Table 1.**

Correlations^*^ of FPG with BMI and waist circumference stratified by region, age, and sex.

|  | FPG and BMI^†^ | | |  | FPG and waist circumference^†^ | | |
| --- | --- | --- | --- | --- | --- | --- | --- |
|  | Overall | Young &  Middle-aged  (< 60 years) | The elderly  (≥ 60 years) |  | Overall | Young &  Middle-aged  (< 60 years) | The elderly  (≥ 60 years) |
| Beijing Chinese | | | | | | | |
| Men | 0.14 | 0.18 | 0.10 |  | 0.20 | 0.23 | 0.15 |
| Women | 0.27 | 0.30 | 0.12 |  | 0.31 | 0.31 | 0.14 |
| Overall | 0.22 | 0.26 | 0.11 |  | 0.28 | 0.29 | 0.16 |
| Guangdong Chinese | | | | | | | |
| Men | 0.13 | 0.15 | 0.14 |  | 0.16 | 0.17 | 0.14 |
| Women | 0.16 | 0.18 | 0.13 |  | 0.21 | 0.22 | 0.15 |
| Overall | 0.15 | 0.17 | 0.14 |  | 0.19 | 0.20 | 0.15 |
| American | | | | | | | |
| Men | 0.17 | 0.18 | 0.16 |  | 0.23 | 0.22 | 0.15 |
| Women | 0.18 | 0.23 | 0.16 |  | 0.25 | 0.26 | 0.20 |
| Overall | 0.18 | 0.20 | 0.15 |  | 0.24 | 0.24 | 0.19 |
| *P* for age difference**^‡^** | | < 0.01 | |  |  | < 0.01 | |

^*^ Coefficient of the correlation between FPG, BMI and waist circumference based on Pearson correlation test.

^†^ All the P values for the correlation between FPG and BMI or waist circumference were less than 0.01.

^‡^ The Wilcoxon test was used to test the difference of coefficients between young & middle-aged adults and the elderly.

**Supplementary Table 2.**

Odds ratio^*^ and 95% confidence intervals for the association between obesity and T2DM stratified by region, age, and sex.

|  |  | Overall |  |  |  | Men |  |  |  | Women |  |
| --- | --- | --- | --- | --- | --- | --- | --- | --- | --- | --- | --- |
|  | Young & Middle-aged  (≤59 years) | Young-Old  (60 ~ 74 years) | Old-Old  (≥75 years) |  | Young & Middle-aged  (≤59 years) | Young-Old  (60 ~ 74 years) | Old-Old  (≥75 years) |  | Young & Middle-aged  (≤59 years) | Young-Old  (60, 74 years) | Old-Old  (≥75 years) |
| Overall subjects |  |  |  |  |  |  |  |  |  |  |  |
| n | 45431 | 12904 | 4800 |  | 22184 | 6657 | 2289 |  | 23247 | 6247 | 2511 |
| Overweight | 2.28(2.03, 2.55) | 1.82(1.62, 2.05) | 1.7(1.44, 2.02) |  | 1.92(1.66, 2.22) | 1.75(1.5, 2.03) | 1.67(1.32, 2.11) |  | 2.6(2.17, 3.12) | 1.89(1.58, 2.26) | 1.71(1.34, 2.18) |
| General Obesity | 5.91(5.33, 6.56) | 3.98(3.56, 4.45) | 3.06(2.57, 3.66) |  | 4.59(4.00, 5.28) | 3.87(3.32, 4.51) | 2.84(2.19, 3.69) |  | 7.81(6.68, 9.17) | 4.29(3.64, 5.08) | 3.32(2.6, 4.25) |
| Abdominal Obesity | 4.05(3.74, 4.39) | 2.72(2.49, 2.98) | 2.29(1.97, 2.68) |  | 3.54(3.2, 3.92) | 2.55(2.27, 2.86) | 2.14(1.75, 2.63) |  | 6.44(5.58, 7.47) | 3.94(3.34, 4.66) | 2.99(2.34, 3.87) |
| Beijing Chinese |  |  |  |  |  |  |  |  |  |  |  |
| n | 6120 | 1575 | 454 |  | 3453 | 1054 | 270 |  | 2667 | 521 | 184 |
| Overweight | 4.04 (2.79, 6.02) | 1.26 (0.87, 1.83) | 0.93 (0.49, 1.71) |  | 2.30 (1.49, 3.67) | 1.24 (0.81, 1.92) | 0.7 (0.33, 1.47) |  | 5.78 (2.75, 12.93) | 1.05 (0.49, 2.24) | 1.39 (0.44, 4.23) |
| General Obesity | 8.84 (5.98, 13.34) | 1.82 (1.14, 2.87) | 1.28 (0.56, 2.73) |  | 4.48 (2.86, 7.27) | 1.94 (1.13, 3.28) | 0.96 (0.33, 2.49) |  | 17.98 (8.03, 41.99) | 1.30 (0.45, 3.3) | 2.02 (0.50, 7.03) |
| Abdominal Obesity | 4.76 (3.63, 6.28) | 2.03 (1.46, 2.83) | 1.51 (0.87, 2.66) |  | 3.43 (2.51, 4.73) | 2.04 (1.4, 2.98) | 1.47 (0.75, 2.95) |  | 5.66 (3.09, 10.28) | 2.00 (1.00, 4.04) | 1.48 (0.55, 3.97) |
| Guangdong Chinese |  |  |  |  |  |  |  |  |  |  |  |
| n | 4508 | 1956 | 450 |  | 1913 | 964 | 225 |  | 2595 | 992 | 225 |
| Overweight | 1.92 (1.45, 2.54) | 1.89 (1.38, 2.57) | 1.56 (0.73, 3.17) |  | 1.59 (1.07, 2.37) | 1.77 (1.15, 2.72) | 1.83 (0.47, 6.12) |  | 2.25 (1.52, 3.34) | 2.04 (1.3, 3.23) | 1.35 (0.52, 3.25) |
| General Obesity | 3.67 (2.65, 5.06) | 2.56 (1.65, 3.89) | 2.57 (0.89, 6.47) |  | 2.92 (1.78, 4.68) | 1.77 (0.81, 3.53) | 5.92 (1.43, 21.69) |  | 4.48 (2.87, 6.94) | 3.32 (1.89, 5.71) | 1.13 (0.17, 4.55) |
| Abdominal Obesity | 3.57 (2.78, 4.57) | 2.10 (1.56, 2.8) | 2.38 (1.24, 4.52) |  | 3.08 (2.14, 4.41) | 1.96 (1.26, 3.00) | 2.92 (1.00, 8.27) |  | 4.07 (2.89, 5.73) | 2.31 (1.54, 3.47) | 1.95 (0.85, 4.42) |
| American |  |  |  |  |  |  |  |  |  |  |  |
| n | 34803 | 9373 | 3896 |  | 16818 | 4639 | 1794 |  | 17985 | 4734 | 2102 |
| Overweight | 2.29 (2.00~ 2.62) | 1.62 (1.42, 1.86) | 1.69 (1.4, 2.03) |  | 2.05 (1.73, 2.44) | 1.52 (1.28, 1.83) | 1.63 (1.26, 2.11) |  | 2.49 (2.01, 3.09) | 1.69 (1.38, 2.08) | 1.69 (1.3, 2.21) |
| Overall Obesity | 5.96 (5.29, 6.73) | 3.16 (2.78, 3.6) | 2.92 (2.41, 3.55) |  | 4.94 (4.22, 5.81) | 3.04 (2.55, 3.64) | 2.65 (1.99, 3.53) |  | 7.55 (6.3, 9.14) | 3.46 (2.87, 4.2) | 3.24 (2.5, 4.22) |
| Abdominal Obesity | 3.92 (3.58, 4.29) | 2.18 (1.96, 2.42) | 2.07 (1.75, 2.45) |  | 3.7 (3.31, 4.14) | 2.19 (1.92, 2.49) | 1.98 (1.59, 2.47) |  | 7.14 (5.94, 8.67) | 3.27 (2.67, 4.04) | 2.92 (2.19, 3.94) |

^*^Subjects with normal weight and normal waist circumference were used as referents for general obesity and abdominal obesity, respectively.

**Supplementary Table 3**

Gender disparity of the odds ratio^*^ for the association between obesity and T2DM stratified by region, age, and sex.

|  | Young & Middle-aged  (≤59 years) | | | | Young-Old  (60 ~74 years) | | | | Old-Old  (≥75 years) | | | |
| --- | --- | --- | --- | --- | --- | --- | --- | --- | --- | --- | --- | --- |
|  | Women | Men | ΔOR^†^ | *P*^‡^ | Women | Men | ΔOR^†^ | *P*^‡^ | Women | Men | ΔOR ^b^ | *P* ^c^ |
| Overall subject | | | | | | | | | | | | |
| General Obesity | 7.85(4.63, 14.5) | 3.59(2.62, 6.63) | 2.19 | < .001 | 2.17 (1.45, 3.26) | 2.21(1.64, 2.76) | 0.98 | 0.51 | 2.34(1.71, 2.97) | 2.53(0.82, 2.82) | 0.92 | 0.37 |
| Abdominal Obesity | 5.32(2.74, 10.8) | 3.09(2.36, 4.2) | 1.72 | < .001 | 2.16(1.71, 2.88) | 1.94(1.81, 2.18) | 1.11 | 0.02 | 2.61(1.97, 2.87) | 1.93(1.77, 3.20) | 1.35 | 0.35 |
| Beijing Chinese | | | | | | | | | | | | |
| General Obesity | 13.2(5.23, 1.1e9) | 3.55(2.31, 10.3) | 3.72 | < .001 | 1.36(1.10, 1.50) | 2.08(1.43, 2.21) | 0.65 | 0.02 | 2.29 (1.92, 3.53) | 0.82(0.65, 1.14) | 2.79 | 0.02 |
| Abdominal Obesity | 5.19(1.83, 1.29) | 3.42(2.11, 4.91) | 1.52 | < .001 | 1.71(1.47, 1.91) | 1.92(1.82, 2.13) | 0.89 | < .001 | 2.65 (1.65, 3.71) | 2.58(1.65, 3.21) | 1.03 | 0.81 |
| Guangdong Chinese | | | | | | | | | | | | |
| General Obesity | 4.98(2.99, 8.26) | 3.74(2.30,13.67) | 1.33 | 0.11 | 1.94(1.74, 3.27) | 1.71(1.48, 2.85) | 1.13 | 0.27 | 1.50(1.38, 1.69) | 6.45(2.05, 7.50) | 0.23 | 0.07 |
| Abdominal Obesity | 4.65(2.44, 11.18) | 3.23(2.66,4.44) | 1.44 | 0.01 | 2.15(1.76, 2.31) | 1.85(1.59, 2.21) | 1.16 | 0.52 | 2.19(1.91, 2.59) | 3.15(1.95, 3.27) | 0.70 | 0.57 |
| American | | | | | | | | | | | | |
| General Obesity | 7.97(5.61, 11.04) | 3.57(3.04, 4.30) | 2.23 | < .001 | 3.20(3.05, 3.46) | 2.66(2.39,3.11) | 1.20 | 0.008 | 2.97(2.86,3.10) | 2.79(2.59, 2.81) | 1.06 | 0.01 |
| Abdominal Obesity | 5.41(4.63, 8.24) | 2.92(2.12, 4.02) | 1.85 | < .001 | 3.09(2.92, 3.74) | 2.05(1.89,2.15) | 1.51 | 6.1e-5 | 2.85(2.66,2.89) | 1.79(1.77, 1.91) | 1.59 | 0.03 |

^*^ Odds ratio was estimated by logistic regression with the SWAN algorithm, stratified by region, age, and sex, described by median value and the interquartile ranges.

^†^ ΔOR= OR _in women_/OR _in men_

^‡^ *P* for gender disparity: The difference of odds ratio between women and men in each age group was tested by the Wilcoxon test.

**Supplementary Table 4**

Life expectancy (year) among USA, China, Chinese Beijing, and Chinese Guangdong Province.

| Year | USA^a^ | | |  | China* | | | | | | | | | | | |
| --- | --- | --- | --- | --- | --- | --- | --- | --- | --- | --- | --- | --- | --- | --- | --- | --- |
|  | Overall | | |  | Overall ^b^ | | |  | Chinese Guangdong ^c^ | | |  | Chinese Beijing ^d^ | | |  |
|  | Total | Male | Female |  | Total | Male | Female |  | Total | Male | Female |  | Total | Male | Female |  |
| 2010 | 78.7 | 76.2 | 81.0 |  | -- | -- | -- |  | 76.49 | 74.00 | 79.37 |  | 80.1 | 78.28 | 82.21 |  |
| 2011 | 78.7 | 76.3 | 81.1 |  | -- | -- | -- |  | -- | -- | -- |  | -- | -- | -- |  |
| 2012 | 78.8 | 76.4 | 81.2 |  | -- | -- | -- |  | -- | -- | -- |  | -- | -- | -- |  |
| 2013 | 78.8 | 76.4 | 81.2 |  | -- | -- | -- |  | -- | -- | -- |  | -- | -- | -- |  |
| 2014 | 78.9 | 76.5 | 81.3 |  | -- | -- | -- |  | -- | -- | -- |  | 81.81 | -- | -- |  |
| 2015 | 78.7 | 76.3 | 81.1 |  | 76.34^b^ | 73.64 | 79.43 |  | 77.24 | -- | -- |  | 81.95 | 79.81 | 84.16 |  |
| 2016 | 78.7 | 76.6 | 81.1 |  | -- | -- | -- |  | -- | -- | -- |  | 82.03 | 79.83 | 84.31 |  |
| 2017 | 78.6 | 76.1 | 81.1 |  | -- | -- | -- |  | -- | -- | -- |  | 82.15 | 79.98 | 84.41 |  |
| 2018 | 78.7 | 76.2 | 81.2 |  | -- | -- | -- |  | -- | -- | -- |  | -- | -- | -- |  |

**^a^** Data come from National Center for Disease Control and Prevention (<https://www.cdc.gov/nchs/data/hestat/life-expectancy/life-expectancy-2018.htm>).

^b^ Data come from National Bureau of Statistics (<https://data.stats.gov.cn/easyquery.htm?cn=C01&zb=A0304&sj=1995>).

^c^ Data come from a statistical website of Guangdong government (<http://stats.gd.gov.cn/>).

^d^ Data come from The People’s Government of Beijing Municipality (<http://www.beijing.gov.cn/>).

* Some Chinese data were not available because these data were not published by government.


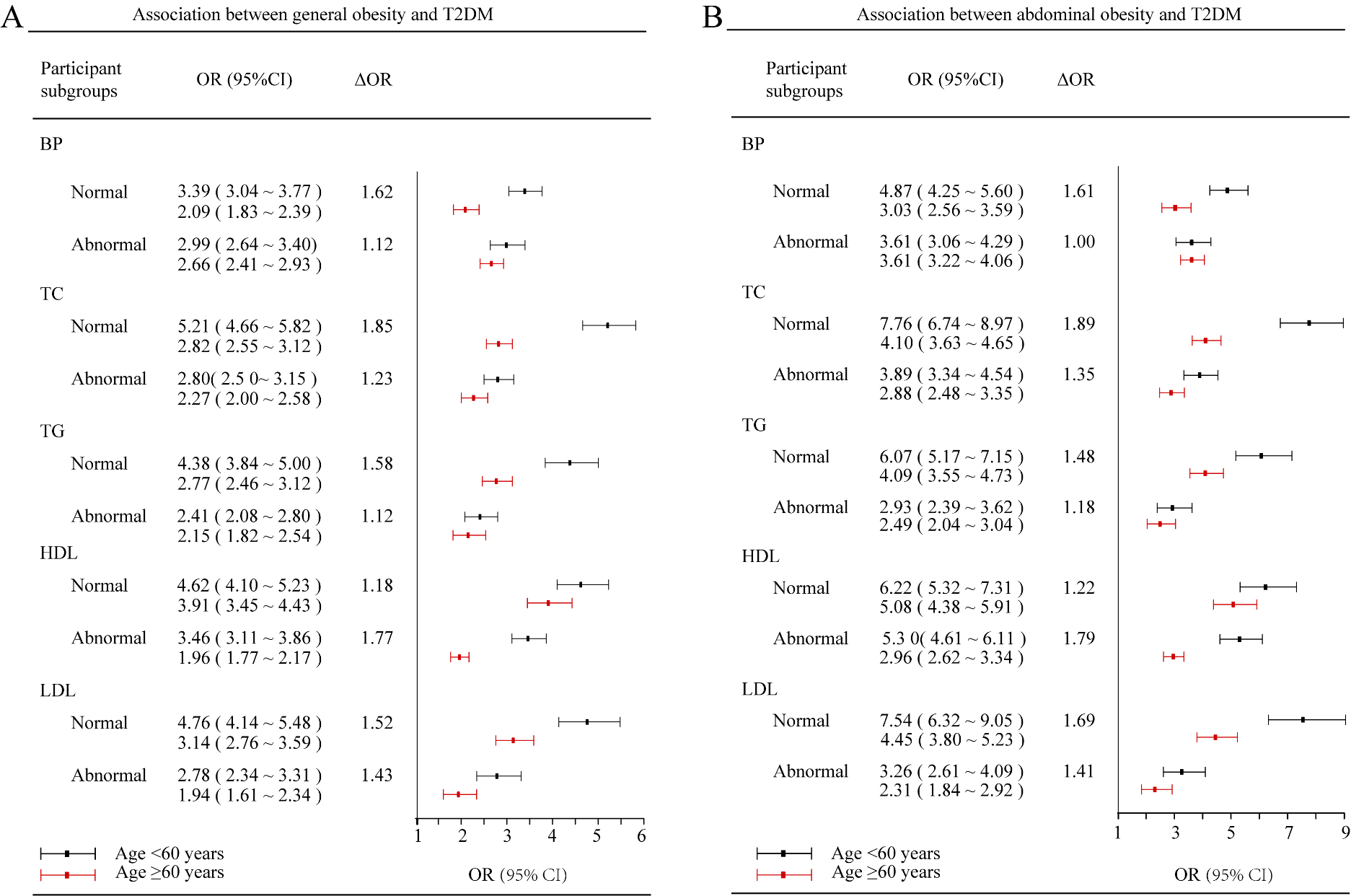


**Supplementary Figure 1.** Sensitivity analyses among metabolic factors for the association between T2DM with both general obesity (A) and abdominal obesity (B). OR (95%CI), odds ratio (95% confidence interval); BP, blood pressure; TC, total cholesterol; TG, triglyceride; HDL-c; high-density lipoprotein cholesterol; LDL-c, low-density lipoprotein cholesterol. Abnormal of BP was diagnosed as systolic blood pressure/diastolic blood pressure ≥ 130/85 mmHg; abnormal TC, ≥5.17mmol/L; abnormal TG, ≥ 1.70 mmol/L; abnormal HDL-c, < 1.03 mmol/L for men and < 1.30 mmol/L for women; LDL-c, > 3.3 mmol/L. Black bar and red bar represent ORs and 95%CI in the young & middle-aged group and the elderly, respectively. ΔOR = OR _for young & middle-aged subjects_/OR _for the elderly._


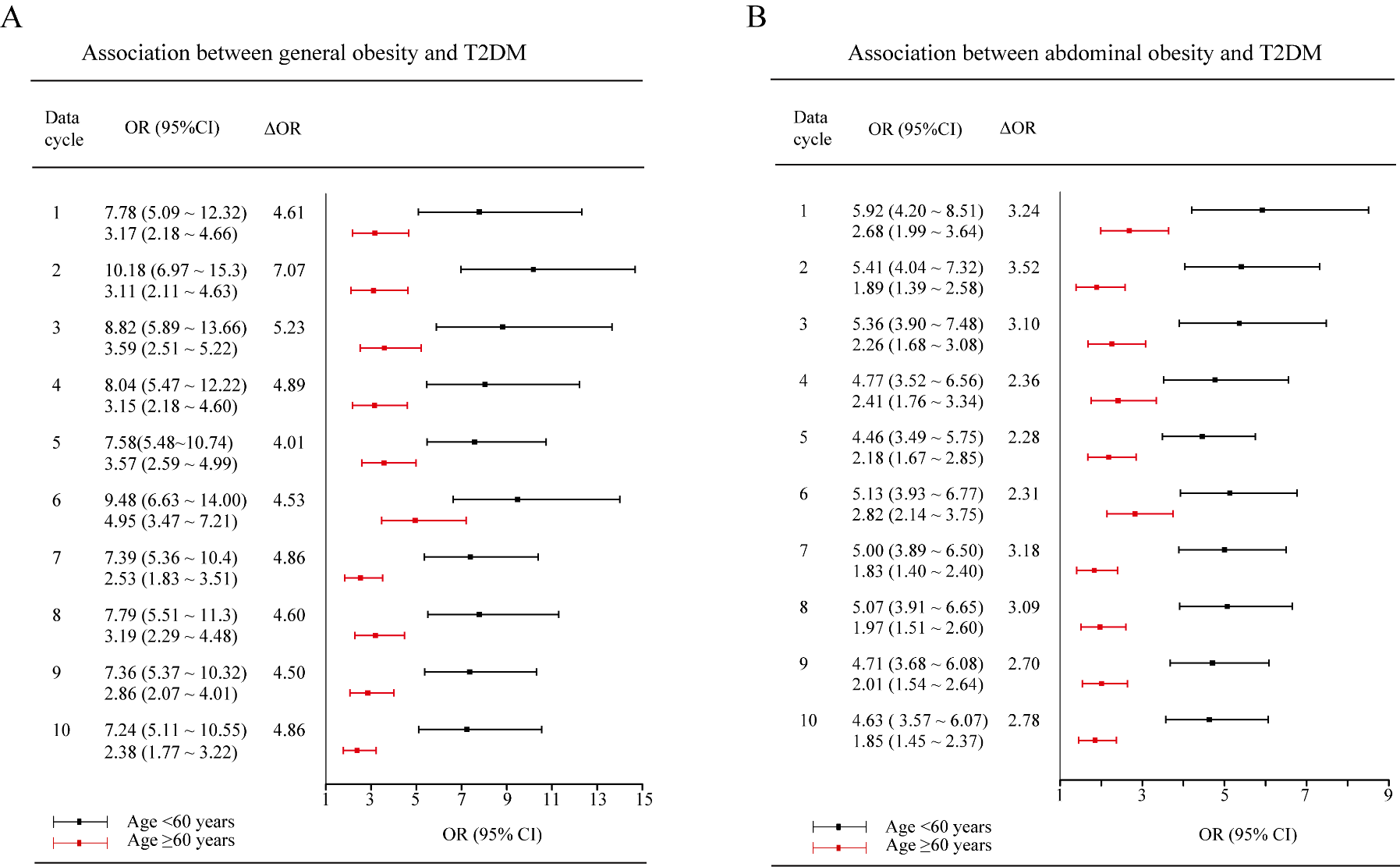


**Supplementary Figure 2.** Sensitivity analyses among different data cycles of the NHANES for the association between T2DM with both general obesity (A) and abdominal obesity (B). NHANES, the National Health and Nutrition Examination Survey from 1999 to 2018 with 10 cycles of investigation and one cycle performed every two years; OR (95%CI), odds ratio (95% confidence interval). Black bar and red bar represent ORs and 95%CI in the young & middle-aged group and the elderly, respectively. ΔOR = OR _for young & middle-aged subjects_/OR _for the elderly_.
